# Learning to read and interpret tattoos

**DOI:** 10.1007/s12024-025-00940-w

**Published:** 2025-01-16

**Authors:** Roger W. Byard

**Affiliations:** Adelaide School of Biomedicine, The University of Adelaide, Level 2, Room N237, Helen Mayo North, Frome Road, Adelaide, South Australia 5005 Australia

**Keywords:** Tattoos, Identification, Origin, Family, Political affiliation, Prison

## Abstract

Tattooing has been a facet of many civilizations and cultures for millennia with a recent resurgence in popularity in many Western countries. The reasons for tattooing are diverse ranging from simple decorative designs to enforced tattooing of concentration camp inmates. In a forensic context tattoos are frequently observed and may play a role in some cases of identification, even after decomposition, incineration or dismemberment. More broadly however, tattoos can provide significant information on a decedent’s name and age, country or region of origin, religion, names of family members and friends, pet ownership, political affiliations, sporting and recreational activities, military service, gang memberships, drug usage and medical data. Thus, careful reading of tattoos at the time of post mortem examination can sometimes be a very productive exercise delivering background material on a decedent that may not have been provided in police reports.

## Introduction

Tattooing refers to the introduction of indelible pigment under the skin to create messages, images or designs. The word derives from the Polynesian *tatau*, however it is a practice that has occurred for millennia among many different and widely dispersed cultures. Although in Western cultures in past centuries it had an association with criminal elements, societal outcasts and psychiatric illness [1, 2] it has recently gained great popularity, resulting in an estimated 10–16% of adolescents and young adults having some form of tattoo, compared to 3–9% of the general population [3, 4]. A review of 100 consecutive adult forensic cases aged 18 years and above examined by the author in 2024 in South Australia (excluding cases with marked decomposition or incineration), however, revealed a much higher number of 29/100 cases (29%) in a coronial population.

While the negative stereotypical associations now do not generally apply due to the numbers of people getting tattooed, sometimes at multiple sites [5], certain tattoos such as those espousing antisocial messages are still associated with significantly higher rates of unnatural and violent deaths [6, 7]. This, of course has to be tempered by the realisation that a symbol such as a swastika has completely different meanings in Western compared to Buddhist societies [8].

As the nature of tattooing has been explored in detail elsewhere [9–11] this report will not go into technical and stylistic details but will focus instead on how the interpretation of tattoos in the mortuary can assist in gaining a greater understanding of the back ground, political and religious beliefs, interests and family members of a particular decedent as well as sometimes giving clues as to the manner of death.

## Purpose

The reasons for tattoos in contemporary settings vary greatly ranging from insignias of gang membership and commemoration of a prison term, to simple artistic designs, expressions of political beliefs/philosophy or simply humorous images or texts [9]. In a forensic context tattoos are often of great use in identifying dismembered, decomposed or burnt individuals. One of the most notorious cases in Australia involved the recognition of a tattooed arm that was disgorged by a tiger shark after it had been captured and placed in a Sydney aquarium. The arm came from a murder victim who had been dismembered and dumped at sea [12].

## Learning from tattoos

Tattoos should be regarded as the pathologist’s friend given the amount of interesting and potentially useful information that they may have recorded.

### Identity

In addition to using tattoos for identification or exclusion by relatives and close friends, on occasion tattoos may include the decedent’s first and/or family names and birth date. Both may clearly provide a very useful start for the identification process if other information is lacking. Their presence or absence has been used in courtrooms since the nineteenth century to attempt to establish identity [13, 14].

### Origin

The country or region of origin of a decedent or cultural group may be surmised from images of national flags, national cultural figures or sometimes even the name or map of a country. As this may be in unlikely places such as the soles of the feet an examination of all skin surfaces is required. Messages may be written in a language that the examiner is not familiar with, particularly if the decedent is a recent refugee or displaced person. Reverse image search is a way of utilizing the internet to quickly identify particular languages and characters. A caveat is the recent use of Chinese calligraphy in some Western tattoos.

Unusual locations of tattoos such as on the buccal mucosa may indicate a specific country such as Eritrea or Ethiopia where this practice occurs [15]. Similarly, distinctive traditional designs in a country such as India may identify an individual from a particular tribal group and/or region [16].

### Religion

Imagery of orthodox crosses may suggest a background in Eastern Europe or Russia and swastikas may be found in Buddhist populations. Images of crosses and Christ or the Virgin Mary are most likely in someone with a Christian background.

### Family and friends

Spouses names with wedding dates are a form of commemorative tattoo and may be augmented with the names of children and their birth dates. The dates of the deaths of relatives or close friends may also be recorded providing clues as to family and social networks. So-called anniversary suicides refer to a suicide that occurs on a particularly important anniversary, often the death of a loved one. As the date of death may be inscribed in a tattoo, death proximate to this in a following year may give an indication as to what may have occurred [17].

### Military service

Previously primitive naval tattoos were often found in sailors and many soldiers from WWII often had simple commemorative tattoos for particular units that they served with, or battles where they had fought. This is also common amongst more contemporary combatants and may include both their names and regimental/service numbers. Specific tattoos to record blood type, ‘*Blutgruppentätowierung*‘, were located on the the inside of the left arm near the axilla of members of the *Waffen-SS* in Nazi Germany. Such tattoos were later used as evidence of membership of the SS in post war tribunals and courts. Other soldiers including in the US military may also have blood group tattoos [18].

### Political affiliations

Probably the best recognised tattoos with political affiliations in Western countries involve swastikas and white supremacist material. In Australia the Eureka flag is often tattooed for this purpose. Other political associations that may be tattooed include party and union emblems.

### Occupation

Clues to this may be gleaned from workplace logos that are sometimes tattooed by factory workers who have been with the one industry for a long time or from union or guild badges and membership numbers [19].

### Pet ownership

Given the high quality of modern fine line tattoos owners of pets sometimes have their animals memorialised by tattoos, or by a paw mark with the animal’s name.

### Antisocial activities/Prison terms

Antisocial tattoos take a variety of forms ranging from simple expletives to self-deprecatory texts and existential questions as to the moral character of God (the later usually phrased in more basic terms using four letter words). Individuals with these types of tattoos are known to be at higher risk of violent and unnatural deaths in a forensic setting [7].

Prison tattoos tend to be monochromatic and primitive, often with an antisocial focus. One tattoo that used to be seen in South Australia was that of the image of a pig with the letters ‘ACAC’ (all cops are *****). This virtually always indicated that a decedent had been in prison and that his/her fingerprints would therefore be on file. Prison tattoos have been taken to the extreme in Russia where extensive tattoos of felons have recorded crimes, sentences, and the dates and places of institutional incarceration [20].

Specific gangs may also use tattooing to identify members such as the Pachuco street gang in California whose members have a cross between the first finger and thumb. *Yakuza* gang members in Japan keep their full body tattoos concealed and do not have tattoos of the hands, neck, head or face [11, 21]. Biker gangs on the other hand tend to display their tattoos that include club and motor cycle symbols and names such as ‘Harley Davidson’.

### Hobbies/sporting interests

Many followers of sports teams, particularly high profile local or national teams commemorate their teams, specific players and any finals victories with tattoos. This may give an indication of a particular city or state where the person either grew up or currently lives.

### Motor cycle and car club membership

Individuals who have a particular attraction to certain brands/makes of vehicles may be involved in non-gang club activities and may have manufactures logos and names tattooed.

### Drug taking

Those involved in the illicit drug scene may have tattoos showing their drug of preference such as marihuana leaves or syringes. These sorts of tattoos indicate that toxicological studies may produce useful post mortem information.

### Medical history and preferences

On occasion those with significant medical problems may elect to have this information tattooed in a prominent location such as the anterior torso rather than depending on a medicalert bracelet being found and read [22]. Information may also include blood grouping and specific diseases or allergies [18]. Evidence of previous radiotherapy indicating cancer might be in the form of strategically placed marker dots. Certain individuals who do not want to be resuscitated in the event of a collapse may also have this information tattooed on their chest where it will be clearly seen by any who are tempted to perform cardiopulmonary resuscitation.

## Conclusion

Careful evaluation of tattoos during forensic examinations can, therefore, provide important information that may include the name and age of the decedent, the country or region of origin, religion, names of family members and friends, pet ownership, political affiliations, sporting and recreational activities, military service, gang memberships, drug usage and medical information. Despite the usefulness of sophisticated DNA technology in identification, sometimes the simple reading of tattoos may provide highly important information rapidly. This was certainly the case in the Snowtown serial killings in Adelaide, Australia in the 1900’s where identification by tattoos was more useful than laboratory-based methods. Learning to appreciate, read and interpret tattoos may be a somewhat old fashioned, but non-the-less useful, forensic skill.
